# Assessment of receptor occupancy-over-time of two dopamine transporter inhibitors by [^11^C]CIT and target controlled infusion

**DOI:** 10.3109/03009734.2011.563878

**Published:** 2011-04-12

**Authors:** Olof Eriksson, Bengt Långström, Ray Josephsson

**Affiliations:** ^1^Department of Radiology, Oncology and Radiation Sciences, Division of Radiology, Uppsala University, Uppsala, Sweden; ^2^Department of Biochemistry and Organic Chemistry, Uppsala University, Uppsala, Sweden; ^3^Clinical Imaging Unit, Novartis Pharma AG, Basel, Switzerland

**Keywords:** CCIP, [^11^C]CIT, DAT inhibitor, SRTM, TCI

## Abstract

**Introduction:**

Occupancy-over-time was determined for two dopamine transporter (DAT) inhibitors through modeling of their ability to displace the PET ligand [^11^C]CIT. The tracer was held at a pseudo steady state in a reference tissue by target controlled infusion.

**Methods:**

Rhesus monkeys (*n* = 5) were given [^11^C]CIT and studied with a PET scanner. Tracer uptake in the reference tissue cerebellum was held at a pseudo steady state by use of target controlled infusion. The pharmacokinetics/pharmacodynamics(PK/PD) of [^11^C]CIT was assessed through the simplified reference tissue model (SRTM). Bupropion (*n* = 2) and GBR-12909 (*n* = 2) receptor occupancies were estimated through modeling of their effects on [^11^C]CIT displacement.

**Results:**

There was a high uptake of [^11^C]CIT in striatum, which contains a high DAT density. The reference tissue cerebellum had a comparatively low uptake. The modeling of [^11^C]CIT PK/PD properties in striatum showed high binding potential (BP = 5.34 ± 0.78). Both DAT inhibitors caused immediate displacement of [^11^C]CIT after administration. The occupancy-over-time was modeled as a mono-exponential function, describing initial maximal occupancy (Occ_0_) and rate of ligand–receptor dissociation (k_off_). GBR-12909 showed irreversible binding (k_off_ = 0) after an initial occupancy of 76.1%. Bupropion had a higher initial occupancy (84.5%) followed by a release half-life of 33 minutes (k_off_ = 0.021).

**Conclusions:**

The proposed model can be used for assessment of in-vivo occupancy-over-time of DAT ligands by use of target controlled infusion of [^11^C]CIT. The concept of assessing drug–receptor interactions by studying perturbations of a PET tracer from a pseudo steady state can be transferred to other CNS systems.

## Introduction

Positron emission tomography (PET) is a non-invasive imaging modality where a tracer containing a positron-emitting nuclide (such as ^11^C or ^18^F) can be studied *in vivo* over time. This study describes how a novel system for target controlled infusion (TCI), UIPump, can be used for tracer modeling and assessment of acute dynamics of unlabeled pharmaceuticals. The basic programming of UIPump has been described earlier (1) as well as some of the possible applications (2,3).

[^11^C]CIT is a tracer for studies of aspects of the central nervous system (CNS), which binds with high specificity to the dopamine transporter (DAT), with low non-specific binding to other structures (4). The tracer is therefore suitable for studies of the interaction between DAT and pharmaceutical compounds that affect the CNS. DAT is expressed in high densities in striatum, while some other structures in the brain are almost devoid (such as cerebellum). In this study the pharmacokinetic and pharmacodynamic (PK/PD) properties of [^11^C]CIT in striatum (both putamen and caudate nucleus) have been assessed by compartmental reference tissue modeling.

DAT exerts its effect through re-uptake of the neurotransmitter dopamine from the synaptic cleft, thereby regulating the postsynaptic response (5). Changes in DAT densities have been associated to attention deficient hyperactivity disorder (ADHD) (6) and several neurodegenerative disorders, such as schizophrenia (7). It is also a target molecule for DAT agonists and antagonists. These classes include both therapeutical drugs and psychoactive drugs (such as cocaine and amphetamine) (8). DAT antagonists impair the re-uptake of dopamine, leading to a modulation of the pre- and postsynaptic function both short- and long-term. These synaptic changes can lead to withdrawal symptoms or abuse problems after long exposure to some DAT ligands.

This study compares acute ligand–target interactions immediately after administration by modeling the occupancy-over-time based on the ability of the compound to displace [^11^C]CIT from DAT. To study the immediate displacement of [^11^C]CIT the pharmaceuticals are administered during a PETscan, where tracer levels are kept at a pseudo steady state in a reference tissue (cerebellum) by using TCI. Apart from designed infusion schemes using TCI, pseudo steady state levels of plasma or tissue can also be achieved by a bolus injection followed by constant infusion of tracer, but the time until steady state is usually longer (9–11).

Occupancy of a ligand to a target is usually determined at equilibrium. The immediate ligand–target pharmacodynamics can be difficult to quantify due to low resolution in the assay. The PK/PD and occupancy of a pharmaceutical can be evaluated based on displacement of a PET tracer. However, in this kind of study the patient is often pretreated (before the scan) with the compound to allow it to reach equilibrium in the target tissue. The initial interactions are therefore often not included.

Different DAT-targeted pharmaceutical compounds have different pharmacodynamics and exert short- or long-term effects. The initial occupancy (during the first seconds, minutes, or hours) may or may not affect the subject. One could for example hypothesize that a very rapid onset of high occupancy, combined with high retention, may trigger up-regulation of DAT density and thereby a risk of habituation to the compound. Conversely, a low initial occupancy with rapid clearance from DAT-rich regions yields a lower risk of causing habituation. Therefore, the onset binding and clearance of two compounds to DAT were investigated in this regard.

Bupropion was chosen as a model of a pharmaceutical with low risk of inducing habituation, while GBR-12909 was used as a model for a compound with high risk of inducing habituation (such as cocaine). Bupropion is an inhibitor of DAT, which has been used in the treatment of ADHD and smoking cessation (12). GBR-12909 has high specific antagonistic binding to DAT with long duration and has been investigated as a treatment drug for cocaine addiction (13).

## Materials and methods

### Animals

Five female rhesus monkeys (weight 5.4–9.7 kg; age 15–20 years) were used. The animals were sedated with ketamine (7 mg/kg) before transportation to the PET camera. One venous catheter was applied for tracer administration and one for administration of pharmacological compounds and blood sampling. Propofol was administered intravenously until the animal was anesthetized enough for intubation. After intubation the animal was maintained on sevoflurane (3%–8%) mixed with medical air and artificial ventilation.

Blood samples were taken for estimates of electrolytes, glucose, and hematocrit.Blood losses were compensated for with injections of albumin (50 mg/mL). Ringer-acetate (0.5 mL/kg/h) was infused during the whole experiment. Body temperature, heart rate, ECG, pCO_2_, pO_2_, SaO_2_, and blood pressure were monitored throughout the PET study.

Experiments were approved by the local Ethics Committee for Animal Research and performed in accordance with local institutional and Swedish national rules and regulations.

### Radiochemistry

[^11^C]CIT was synthesized as described previously (4). The radiochemical purity of each batch was greater than 95%.

### Infusion system

The TCI system UIPump consists of a personal computer and an infusion pump. For this study a programmable Univentor 864 Infusion Pump (AgnThos, Lidingo, Sweden) was used. The UIPump program compares the target concentration curve with a model of the compound kinetics after a fast bolus injection and calculates the infusion scheme necessary for compensating the wash-out of the compound to generate a steady state of the compound in a specific tissue. In this study the TCI system was used to generate a steady state in the reference tissue cerebellum.

### PET protocol

The PET imaging studies were performed using a SHR 7700 camera (Hamamatsu Photonics, Hamamatsu, Japan). The scanner has a 14.3 × 11.4 cm field of view, and the scanner is capable of an axial and trans-axial resolution of 2 mm. All scans were performed in 2D. An 18.5 MBq ^68^Ge rotating point source was used to acquire normalization and blank scans. The attenuation scans were performed in coincidence mode for 20 minutes. Dead time correction, random correction, and scatter correction were performed for all scans. Images were reconstructed by using 2D filter back projection.

All scans were performed with infusion of the radiotracer calculated by UIPump, aiming for steady state in cerebellum. The base-line scan was performed over 100 minutes (five animals). The second scan was performed over 110 minutes where a DAT ligand, bupropion (*n* = 2) or GBR-12909 (*n* = 2), was administered after 60 minutes at a pharmacological dose.

All images were analyzed in IDA (Scanditronix AB, Uppsala, Sweden). Regions of interest (ROIs) were drawn over striatum and cerebellum in several transversal slices in each subject. Each collection of ROIs was combined into a volume of interest (VOI), and time–activity curves (TACs) were generated from each VOI. Standardized Uptake Values (SUV) values were calculated by using only the amount of radioactivity administrated by the initial short bolus. This procedure resulted in apparently high SUV values, as the initial bolus contributed on average only 23% of the total administered tracer volume/activity (the remaining 77% consisting of discrete subsequent infusions).

### Modeling of [^11^C]CIT uptake

The [^11^C]CIT uptake in striatum was investigated by implementing the TCI system to achieve a pseudo steady state in the reference tissue cerebellum. PK/PD parameters R_1_, k_2_, binding potential (BP), and distribution volume ratio (DVR) were determined by fitting the striatal TACs to the simplified reference tissue model (SRTM) described by Lammertsma et al.(14). Cerebellum was used as reference region due to its low expression of DAT. The DVR in the reference region was set to 1, assuming BP_cerebellum_ = 0. The model was implemented by the MatLabprogram Rz (in-house, Uppsala Imanet AB, Uppsala, Sweden).

### Assessment of occupancy-over-time

The DAT inhibitors bupropionhydrochloride (Sigma-Aldrich, St Louis, MO, USA) or GBR-12909 (RBI, Natick, MA, USA) were dissolved in 5 mL 0.1 M NaCl. They were then administered intravenously as a bolus (5 mg/kg) 60 minutes after administration of radiotracer.

SRTM is based on the full reference tissue model (15). In the full model the target tissue is described as two compartments, C_free_ and C_bound_. Several rate constants (k_2_, k_3_, and k_4_) determine the tracer exchange between the compartments. The rate constant k_2_ determines wash-out from the tissue, while k_3_ and k_4_ represent tracer–target binding and release, respectively. In SRTM, the ratio k_3_/k_4_ is denoted as BP and the two compartments combined into one. The distribution volume ratio (DVR = BP + 1) can be considered to specify the ratio between bound and free tracer.

When a DAT inhibitor displaces [^11^C]CIT in the striatum, the tracer concentration in the region will decrease due to active transport through the cellular membrane. Only free tracer is available for wash-out through k_2_. Therefore, an increase in wash-out must be due to an increase in free tracer (or a decrease in BP which determines DVR). Decrease in BP is here assumed to equal a decrease in available receptors for the tracer (the apparent k_3_ becomes lower) due to pharmaceutical binding to DAT. The occupancy of all available DAT (in %) can then be calculated from the apparent decrease in BP (BP_apparent_) (equation 1) due to [^11^C]CIT displacement and wash-out.

(Eq.1) $$\begin{array}{l} Occupancy=\frac{(BP-BP_{apparent})\;*\;100}{BP},\\ where\;BP_{apparent}=k_{3apparent}/k_{4} \end{array}$$

The change in tracer concentration in the striatum can be described by a one-tissue compartment (equation 2). The term α is equal to the plasma concentration of tracer (C_p_) multiplied by the unknown rate constant K_1_. C_p_ is assumed to be proportional to C_cerebellum_ and therefore constant during the displacement phase; α is subsequently treated as a constant.

(Eq.2) $$\begin{array}{l} \frac{dC}{dt}=\alpha -k_{2}*\frac{C(t)}{1+(BP_{apparent})},\\ where\;\alpha =C_{p}*K_{1} \end{array}$$

Occupancy of each inhibitor to DAT was described as a mono-exponential function (equation 3). The constants Occ_0_ and k_off_ denote initial occupancy (in %) and ligand–target dissociation (in min^-1^), respectively. There is always an ‘absorption phase’ (usually very short) before the maximal initial occupancy is reached. The absorption can be described by an additional exponential term in the function, but it is not included here for simplicity.

(Eq.3) $$Occupancy=Occ_{o}*\;e^{[(k_{off}\text{] }*\text{ t)}}$$

Equations 1–3 were combined into equation 4, which was fitted discretely to the experimental data for each DAT inhibitor in MatLab. Best fit was determined iteratively through residual sum of squares.

(Eq.4) $$\frac{dC}{dt}=\alpha -k_{2}*\;\frac{C(t)}{1+\left(\frac{BP\;*\;(100-Occ_{o}*\;e(k_{off}*t)}{100}\right)}$$

## Results

### Bolus curve acquisition

**Figure 1.**
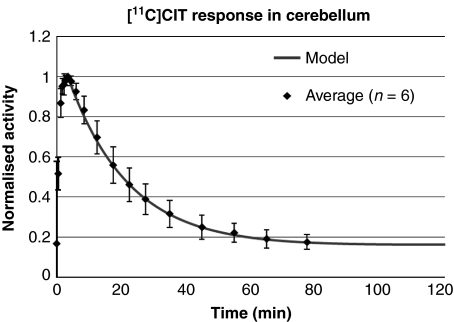
The response of [^11^C]CIT in cerebellum was modeled from TACs from six previous experiments where the tracer was administered as an intravenous bolus. Error bars indicate standard deviation. The model curve was used to calculate the infusion scheme required to achieve pseudo steady state of tracer in cerebellum.

The UIPump bolus input curve was modeled after TACcurves over cerebellum from earlier experiments (*n* = 6) where [^11^C]CIT was administered intravenously (data not shown). The curve in Figure 1 is the mono-exponential curve (t_½_ = 13.2 min; plateau at 16.2% of initial value) with best fit to the individual bolus responses in previous experiments.

### Base-line acquisition

**Figure 2.**
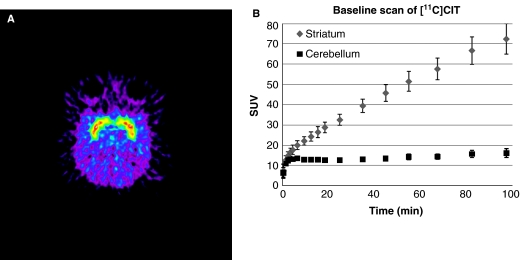
Target-controlled infusion to reach pseudosteady state of [^11^C]CITin cerebellum. As expected, there was high uptake localized to striatum, as shown in a trans-axial projection of a representative subject (A). The pseudosteady state of [^11^C]CITin cerebellum resulted in elevated, close to irreversible striatal uptake (B). Error bars indicate SEM (*n* = 5).

The bolus input curve was used to program an infusion scheme to reach a pseudo steady state of [^11^C]CIT in the cerebellum. Uptake in striatum (Figure 2A) increased close to linearly during the entire scan under these conditions, indicating that the interaction between tracer and target was almost irreversible (Figure 2B).

### Modeling of [^11^C]CIT uptake

Results from fitting SRTM to striatum using cerebellum as a reference region are presented in Table I. Each subject was modeled individually. Parameters are given as averages ± SEM. The estimated k_2_ rate constant values (mean k_2_ = 0.10 ± 0.1) confirmed that [^11^C]CIT has close to irreversible binding properties in striatum. BP was high in all subjects (mean BP = 5.34 ± 0.78); especially subject 3 (BP = 8.14) which is the source of the majority of variation in SEM.

**Table I.** Kinetic parameters for [^11^C]CIT PK/PD in striatum determined from the simplified reference tissue model. The majority of variation in BP and DVR is due to the elevated values in subject 3.

Parameter	Subject 1	Subject 2	Subject 3	Subject 4	Subject 5	Average	SEM
R_1_	0.58	1.09	1.47	1.17	0.84	1.03	0.15
k_2_	0.13	0.08	0.12	0.07	0.07	0.10	0.01
BP	3.85	4.18	8.14	4.69	5.87	5.34	0.78
DVR	4.85	5.18	9.14	5.69	6.87	6.34	0.78
DVR ref	1	1	1	1	1	1	-

### Assessment of occupancy-over-time

**Figure 3.**
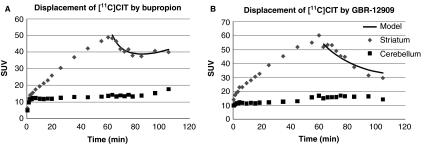
Intravenous administration at 60 minutes of either bupropion 5 mg/kg (A) or GBR-12909 5 mg/kg(B). Infusion is aimed at pseudo steady state of [^11^C]CIT in cerebellum. Best fit occupancy models for each DAT inhibitor are presented as black lines.

Both bupropion and GBR-12909 immediately displaced [^11^C]CIT in striatum (Figure 3). There was no decrease in the cerebellum, confirming that the uptake of [^11^C]CIT in the reference tissue was non-displaceable (uptake consists entirely of free or non-specific binding).

Bupropion and GBR-12909 occupancy could be modeled according to equation 4 above (Table II). GBR-12909 showed lower initial occupancy than bupropion(76.1% compared to 84.5%). The retention of GBR-12909 was very high, however, with a k_off_ = 0 as best fit. This indicates completely irreversible binding to DAT, at least within the first hour investigated here. If this assessment is true or a limitation of the model will be discussed below. Bupropion–DAT dissociation was determined as k_off_ = 0.021, or as having a retention half-life of 33.0 minutes.

**Table II.** Occupancy model parameters describing the initial pharmacodynamics of bupropion and GBR-12909. Best fit was obtained by minimizing the residual sum of squares (RSS).

Parameter	Unit	Bupropion	GBR-12909
Occ_0_	%	84.5	76.1
k_off_	min^-1^	0.021	0.0
Model fit (RSS)		0.33	1.68

## Discussion

This study aimed to show that the technique of holding a PET tracer at a pseudo steady state in a reference tissue by TCI can be used to determine early changes in receptor occupancy of unlabeled pharmaceuticals at their site of action, as well as quantifying the rate of offset (k_off_).

The PK/PD of the radiotracer [^11^C]CIT was assessed by a simplified reference tissue model. The level of the tracer in the cerebellum was held constant through target controlled infusion by UIPump (using a cerebellum input function modeled from several previous experiments). Preferably, the target tissue should be held at steady state, but this was not possible in this specific case since there is virtually no clearance of [^11^C]CIT from the striatum due to its very strong binding to DAT. The high BP in striatum (5.34 ± 0.78) obtained from the model is consistent with this reasoning.

When studying interactions between an unlabeled compound and a target *in vivo* (for example in an animal model or in patients) with PET, the PK/PD of the compound is essentially determined indirectly through the displacement of the administered PET tracer. Often, the unlabeled compound is administered some time before the tracer to reach equilibrium. In that case, the initial kinetics and dynamics have already occurred at the time of tracer administration and PET scanning. This study showed that all phases of the PK/PD of a compound (including the initial kinetics) can be investigated indirectly through perturbations of a PET tracer at pseudo steady state in a reference tissue.

The perturbations of the radiotracer steady state due to an unlabeled compound were quantified as changes due to ligand–target occupancy-over-time. The occupancy model omitted the absorption phase entirely and focused solely on the clearance phase. To study the absorption phase, which can be very rapid, the model probably would need to have resolution and robustness enough to determine large changes during the first minute(s).

Previous PET studies in humans have determined bupropion occupancy of DAT to 26% (steady state dose regimen, 150 mg dose 3 hours prior to scan (16)) and 14% (steady state dose regimen, 150 mg dose 8–15 hours prior to scan (17)). In this study bupropion was found to have a high initial occupancy (84.5%) and a retention half-life of 33 minutes (5 mg/kg dose in rhesus monkey). The clearance would reduce the initial occupancy to the previously reported values in 1–2 hours.

GBR-12909 showed high initial occupancy of 76.1% combined with zero release during the first 60 minutes. The occupancy is consistent with a previous PET study in rhesus monkey, where doses of 3 and 10 mg/kg bupropion 90 minutes later produced DAT occupancies of 53% and 72%, respectively (18). The very low release seen here indicates a long-term occupancy, which has been previously seen in both rats and rhesus monkeys (13). The optimal model fit for GBR-12909-induced displacement was not ideal. This may be due to the fact that the absorbance phase was omitted from the model, since the fit correlates best with a negative k_off_. A k_off_ below zero is of course impossible in a biological system, but it indicates that occupancy rose slightly during the first hour after initial displacement.

The model for habituation hypothesized in the introduction was based on the initial occupancy (Occ_0_) and the concentration- and time-dependent release (k_off_). For these two compounds Occ_0_ differed very little. Bupropion, which has a steady state dose regimen at low occupancies, was found to have a higher Occ_0_ than GBR-12909. Both compounds exert noticeable initial binding to DAT.

In contrast k_off_ differed greatly between the compounds where bupropion was released with a short half-life (only 25% of the binding remains after approximately 1 hour). GBR-12909 essentially binds irreversibly which could lead to a prolonged DAT inhibition resulting in more prominent downstream regulation to the striatal dopamine system than that seen after bupropion administration.

In conclusion, the proposed model can be used for assessment of *in vivo* occupancy-over-time of DAT ligands by use of target controlled infusion of [^11^C]CIT. The concept of assessing drug-receptor interactions by studying perturbations of a PET tracer from a pseudo steady state can be transferred to other CNS systems.
